# Intensive care unit cardiac arrest among very elderly critically ill patients – is cardiopulmonary resuscitation justified?

**DOI:** 10.1186/s13049-024-01259-1

**Published:** 2024-09-11

**Authors:** Markus Haar, Jakob Müller, Daniela Hartwig, Julia von Bargen, Rikus Daniels, Pauline Theile, Stefan Kluge, Kevin Roedl

**Affiliations:** 1 Department of Intensive Care Medicine, University Medical Center Hamburg-Eppendorf, Martinistraße 52, 20246 Hamburg, Germany; 2 Department of Anaesthesiology, Tabea Hospital, Kösterbergstraße 32, 22587 Hamburg, Germany

**Keywords:** Nonagenarians, Very elderly, Cardiac arrest, Multiple organ failure, Post-cardiac arrest organ failure, Intensive care unit, In-hospital cardiac arrest

## Abstract

**Introduction:**

The proportion of very elderly patients in the intensive care unit (ICU) is expected to rise. Furthermore, patients are likely more prone to suffer a cardiac arrest (CA) event within the ICU. The occurrence of intensive care unit cardiac arrest (ICU-CA) is associated with high mortality. To date, the incidence of ICU-CA and its clinical impact on outcome in the very old (≥ 90 years) patients treated is unknown.

**Methods:**

Retrospective analysis of all consecutive critically ill patients ≥ 90 years admitted to the ICU of a tertiary care university hospital in Hamburg (Germany). All patients suffering ICU-CA were included and CA characteristics and functional outcome was assessed. Clinical course and outcome were assessed and compared between the subgroups of patients with and without ICU-CA.

**Results:**

1,108 critically ill patients aged ≥ 90 years were admitted during the study period. The median age was 92.3 (91.0–94.2) years and 67% (*n* = 747) were female. 2% (*n* = 25) of this cohort suffered ICU-CA after a median duration 0.5 (0.2–3.2) days of ICU admission. The presumed cause of ICU-CA was cardiac in 64% (*n* = 16). The median resuscitation time was 10 (2–15) minutes and the initial rhythm was shockable in 20% (*n* = 5). Return of spontaneous circulation (ROSC) could be achieved in 68% (*n* = 17). The cause of ICU admission was primarily medical in the total cohort (ICU-CA: 48% vs. No ICU-CA: 34%, *p* = 0.13), surgical - planned (ICU-CA: 32% vs. No ICU-CA: 37%, *p* = 0.61) and surgical - unplanned/emergency (ICU-CA: 43% vs. No ICU-CA: 28%, *p* = 0.34). The median Charlson Comorbidity Index (CCI) was 2 (1–3) points for patients with ICU-CA and 1 (0–2) for patients without ICU-CA (*p* = 0.54). Patients with ICU-CA had a higher disease severity according to SAPS II (ICU-CA: 54 vs. No ICU-CA: 36 points, *p* < 0.001). Patients with ICU-CA had a higher rate of mechanically ventilation (ICU-CA: 64% vs. No ICU-CA: 34%, *p* < 0.01) and required vasopressor therapy more often (ICU-CA: 88% vs. No ICU-CA: 41%, *p* < 0.001). The ICU and in-hospital mortality was 88% (*n* = 22) and 100% (*n* = 25) in patients with ICU-CA compared to 17% (*n* = 179) and 28% (*n* = 306) in patients without ICU-CA. The mortality rate for patients with ICU-CA was observed to be 88% (*n* = 22) in the ICU and 100% (*n* = 25) in-hospital. In contrast, patients without ICU-CA had an in-ICU mortality rate of 17% (*n* = 179) and an in-hospital mortality rate of 28% (*n* = 306) (both *p* < 0.001).

**Conclusion:**

The occurrence of ICU-CA in very elderly patients is rare but associated with high mortality. Providing CPR in this cohort did not lead to long-term survival at our centre. Very elderly patients admitted to the ICU likely benefit from supportive care only and should probably not be resuscitated due to poor chance of survival and ethical considerations. Providing personalized assurances that care will remain appropriate and in accordance with the patient’s and family’s wishes can optimise compassionate care while avoiding futile life-sustaining interventions.

**Supplementary Information:**

The online version contains supplementary material available at 10.1186/s13049-024-01259-1.

## Background

The global demographic shift, characterised by declining fertility and increased life expectancy, is leading to anticipated growth in the absolute number of very old patients, specifically those who are 90 years or older [1]. Consequently, this shift suggests a corresponding increase in the proportion of very elderly patients requiring intensive care treatment. Approximately 15% of critically ill patients in the ICU present at an advanced age of 80 years and above; 1% was observed to be beyond 90 years of age [2–4]. While studies have reported acceptable outcomes among this very elderly patients, debates on limitation of therapy and futility of care remain a subject of considerable debate [3, 5].

The incidence of cardiac arrest (CA) markedly escalates with age [6]. Especially in the population of the very elderly critically ill, the debates around therapy limitations and futility are controversial [3, 7–9]. Notably, around half of CA occurring in hospitals are situated in the intensive care unit (ICU) [10]. Cardiac arrest in the ICU (ICU-CA), represents a specific subgroup of in-hospital cardiac arrest (IHCA) and has been less explored in the existing literature [11–14]. The previously reported incidence of ICU-CA varies greatly (4–78/1,000 admissions) [11, 15–17]. The medical response to such events is distinct given the advantages of continuous monitoring, advanced therapeutic options, and the presence of more healthcare professionals [15]. Currently, the occurrence and outcome of ICU-CA in very elderly critically ill patients remains unclear.

This study aims to provide valuable insights into patterns and implications of ICU-CA among patients aged ≥ 90 years in a large tertiary care university hospital.

## Methods

### Study design, setting and ethics

Data of all adult patients ≥ 90 years consecutively admitted to the Department of Intensive Care Medicine at the University Medical Centre Hamburg-Eppendorf (Germany) between January 2008 and April 2019 were analysed. The department constituted of 12 intensive care units (ICU) and provides care to all critically ill adult patients within the hospital with a maximum capacity of 140 beds. Due to the retrospective nature of the study and anonymised data collection the need for informed consent was waived by the Ethics Committee of the Hamburg Chamber of Physicians.

### Inclusion and exclusion criteria

All adult patients (≥ 90 years) admitted to the ICU were included in the study. All patients < 90 years of age, or patients with incomplete clinical data were excluded.

#### Data collection

Data was collected through electronical patient data management system (PDMS, Integrated Care Manager^®^(ICM), Version 9.1 – Draeger Medical, Luebeck, Germany). The extracted data included age, sex, comorbidities, admission diagnosis, length of ICU- and hospital-stay, outcome, treatment modalities and organ support (invasive mechanical ventilation [IMV], vasopressor, renal replacement therapy [RRT], blood transfusions, etc.), laboratory parameters as well as occurrence and characteristics of cardiac arrest.

### Study definitions and patient management

Cardiopulmonary resuscitation and post-CA care were performed in accordance with the European Resuscitation Council Guidelines [18, 19]. Data was collected prospectively according Utstein-style guidelines [20]. ICU-CA was defined as a cessation of circulation, and therefore, an indication for chest compression and/or cardiac defibrillation in patients who had a pulse and circulation at the time of ICU admission. Patients with prior OHCA/IHCA and re-arrest in the ICU were not considered as ICU-CA. The sustained return of spontaneous circulation (ROSC) was defined as stable circulation for at least 20 min. Survivors were tracked throughout their hospital stay after CA for assessment of survival. Cerebral function, as part of routine clinical care, was assessed by physicians on-site. Cerebral Performance Categories (CPC) were utilised to assess neurological outcomes, with a score of 1 to 2 indicating a favourable outcome, and 3 to 5 indicating an unfavourable outcome. In addition, the aetiology of CA – such as cardiac, pulmonary, or cerebral, as well as sepsis, intoxication, hypothermia, and others –were investigated. Severity of illness was evaluated by the sequential organ failure assessment (SOFA) [21] and simplified acute physiology score (SAPS II) [22] on admission. The Charlson Comorbidity Index (CCI) was calculated asassing the overall health burden [23]. Sepsis and septic shock were defined according to the 2016 Third International Consensus Definition for Sepsis and Septic Shock [24].

### Statistical analysis

Data are presented as absolute numbers with relative frequency or medians accompanied by the interquartile range (IQR). Categorial variables were compared using either Chi-Square-Analysis or Fisher’s exact test. For continuous variables, Mann-Whitney-U-Test was employed.

The statistical analysis was conducted using IBM SPSS Statistics Version 24.0 (IBM Corp., Armonk, NY). Throughout the analysis, a p-value < 0.05 was considered statistically significant.

The study was prepared in accordance with the STROBE (STrengthening the Reporting of OBservational studies in Epidemiology) recommendations [25].

## Results

### Study population

**Fig. 1:**
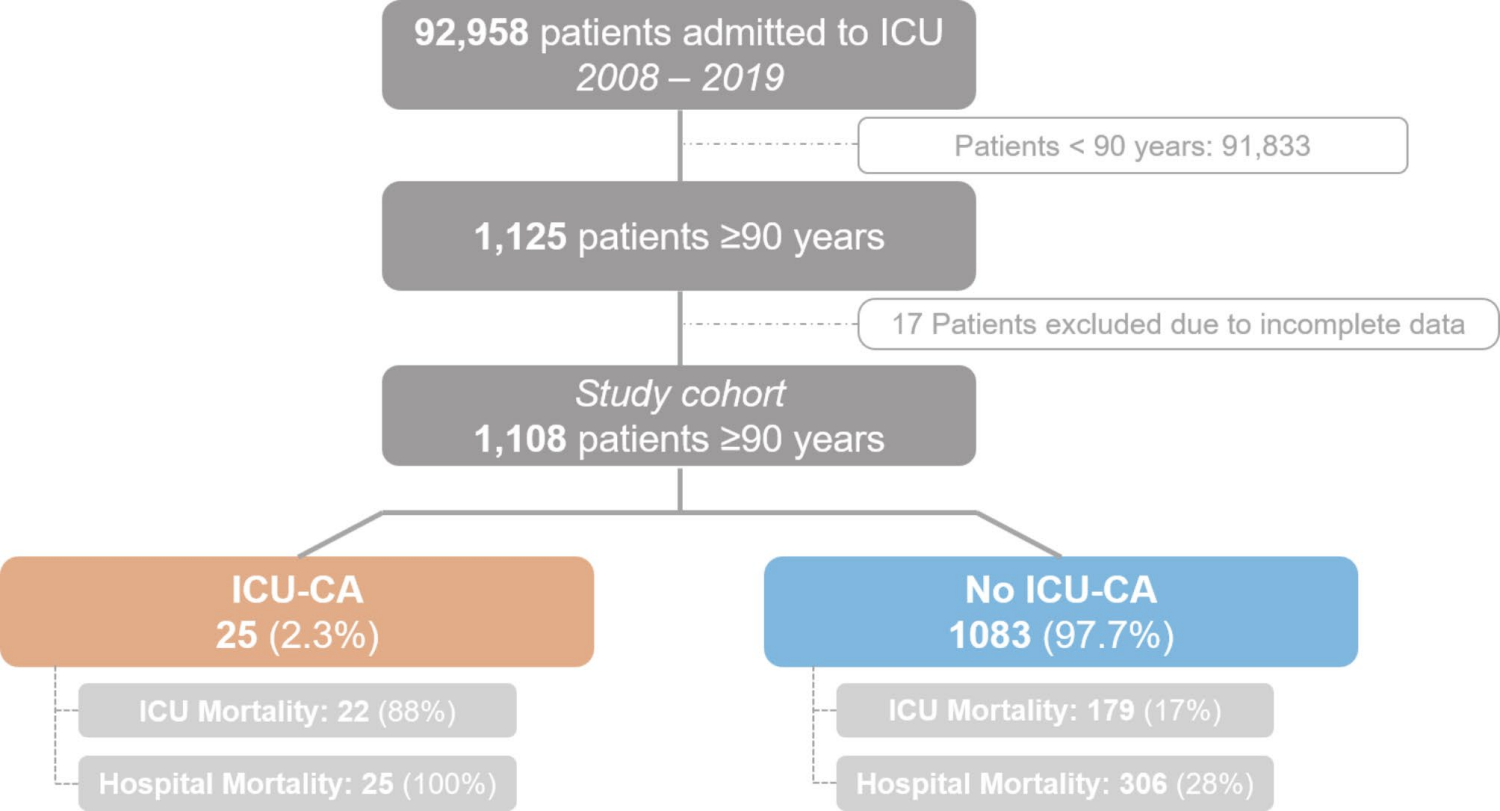
Flow chart of the study

During the study period, a total number of 92,958 patients were admitted. After the exclusion of 17 cases due to incomplete data 1,108 patients ≥ 90 years were identified and included in this study (see Flow-Chart Fig. 1).

The median age of the study population was 92.3 (IQR 91.0-94.2) years and 67% (*n* = 747) were female. The cause of ICU-admission was medical (34%, *n* = 376), elective surgical (37%, *n* = 409) and emergency surgical (29%, *n* = 316). The median CCI was 1 point (0–2). The severity of disease, as represented by the SAPS II and SOFA score, had a median of 36 points (28–47) and 2 points (1–5) on admission, respectively. Vasopressor therapy was necessary in 42% (*n* = 468). Invasive mechanical ventilation was utilised in 33% (*n* = 248) with a median duration of 0.5 days (0.2–1.2). Renal replacement therapy was initiated in 3% (*n* = 31). The rate of tracheostomy was 1% (*n* = 14).

### ICU characteristics in patients with and without ICU-CA

In the total cohort, 2% (*n* = 25) experienced an ICU-CA. Detailed baseline characteristics of patients with and without ICU-CA are presented in Table 2. The mean age of patients with ICU-CA was slightly lower as compared to patients without ICU-CA (91.8 vs. 92.3, *p* = 0.19). Furthermore, as compared to males, females were more likely to experience ICU-CA, although not statistically significant (56% vs. 44%). Comparing ICU-CA to no-ICU-CA, the ICU admission cause was medical (48% vs. 34%, *p* = 0.13), elective surgical (32% vs. 37%, *p* = 0.61) and emergency surgical (20% vs. 29%, *p* = 0.34), respectively. The median CCI was 2 points (1–3) in patients with ICU-CA and 1 point (0–2) in patients without ICU-CA (*p* = 0.54). Additionally, the illness severity as reflected by the SAPS II (54 points [39–65] vs. 36 points [28–46], *p* < 0.001) and SOFA score (5 points [2–9] vs. 2 points [1–5] , *p* < 0.01) was significantly higher on admission as well 24 h after (SOFA: 6 points [IQR: 2–10] vs. 2 points [IQR: 1–4], *p* < 0.001). Vasopressor therapy was initiated in 88% (*n* = 22) and 41% (*n* = 446), respectively (*p* < 0.001). The requirement of invasive mechanical ventilation (IMV) was higher (64% [*n* = 16] vs. 34% [*n* = 373], *p* < 0.01) as well as its required duration was longer (0.8 days [0.4-1.0] vs. 0.5 days [0.2–1.3], *p* = 0.57) in patients with ICU-CA. Renal replacement therapy was initiated in 20% (*n* = 5) and 2% (*n* = 26), respectively (*p* < 0.001). The peak serum lactate levels were higher (4.8 vs. 1.7 mmol/l, *p* < 0.001) and minimum pH values were lower (7.18 vs. 7.36, *p* < 0.001) in the ICU-CA subgroup. Before the hospital stay 76% (*n* = 19) of patients with ICU-CA and 61% (*n* = 657) with No-ICU-CA were living at home, 20% (*n* = 5) and 30% (*n* = 329) were in a nursing facility and 9% (*n* = 94) without ICU-CA were in an assisted living facility.

**Table 1:** Baseline characteristics of patients

Variables	All patients *(n = 1108)*
Age (years)	92.3 (91.0–94.2)
Weight (kg)	65 (55–74)
Height (cm)	165 (160–170)
Female	747 (67)
Primary Admission	
- Medical	376 (34)
- Surgical – planned	409 (37)
- Surgical – emergency	316 (29)
Disease Severity	
Charlson comorb. index, pts.	1 (0–2)
SAPS II – admission (pts.)	36 (28–47)
SOFA – admission (pts.)	2 (1–5)
SOFA – 24 h (pts.)	2 (1–4)
Procedures/Therapies	
Vasopressors	468 (42)
Invasive MV	389 (35)
Duration of MV (days)	0.5 (0.2–1.2)
Renal replacement therapy	31 (3)
Tracheostomy	14 (1)
Outcome	
Duration ICU stay (days)	1.6 (0.9–3.5)
Duration hospital stay (days)	11.0 (7.0–16.6)
Died in ICU	201 (18)
Died in hospital	331 (30)

Data are expressed as n (%) or median (interquartile range – IQR 25/75%)

*Abbreviations*: kg, kilogram; m, meter, SAPS, simplified acute physiology score; SOFA, sequential organ failure assessment; pts, points; MV, mechanical ventilation; ICU, intensive care unit;

**Table 2:** Characteristics of patients with and without intensive care unit cardiac arrest

Variables	Non ICU-CA *(n = 1083)*	ICU-CA *(n = 25)*	*p*-value
Age (years)	92.3 (91.0–94.2)	91.8 (90.9–93.0)	0.19
Weight (kg)	65 (55–74)	65 (60–70)	0.79
Height (cm)	165 (160–170)	170 (165–173)	0.16
Female	733 (68)	14 (56)	0.83
Primary Admission			
- Medical	364 (34)	12 (48)	0.13
- Surgical – planned	401 (37)	8 (32)	0.61
- Surgical – emergency	311 (29)	5 (20)	0.34
Disease Severity			
- Charlson comorb. index, pts.	1 (0–2)	2 (1–3)	0.54
- SAPS II – admission (pts.)	36 (28–46)	54 (39–65)	< 0.001
- SOFA – admission (pts.)	2 (1–5)	5 (2–9)	< 0.01
- SOFA – 24 h (pts.)	2 (1–4)	6 (2–10)	< 0.01
Admission characteristics			
- MAP – adm. (mmHg)	86 (72–102)	79 (63–94)	0.11
- HR – adm. (per min)	80 (65–97)	91 (85–130)	< 0.001
- Temperature – adm. (C°)	36.2(35.5–36.8)	36.5 (35.9–36.9)	0.31
- Vasopressor – adm.	358 (33)	13 (52)	< 0.05
- Invasive MV – adm.	310 (29)	12 (48)	< 0.05
- paO_2_ – adm. (mmHg)	92.7 (73.7–130.8)	117 (79.9–186.0)	0.15
- paCO_2_ – adm. (mmHg)	41.0 (36.4–46.5)	43.9 (40.3–47.7)	0.12
- pH – adm.	7.37 (7.33–7.42)	7.32 (7.27–7.38)	< 0.01
- Lactate – adm. (mmol/l)	1.1 (0.8–1.8)	2.0 (1.5–3.2)	< 0.001
Procedures/Therapies - ICU			
- Vasopressors	446 (41)	22 (88)	< 0.001
- Invasive MV	373 (34)	16 (64)	< 0.01
- Duration of MV (days)	0.5 (0.2–1.3)	0.8 (0.4–1.0)	0.57
- Renal replacement therapy	26 (2)	5 (20)	< 0.001
- Tracheostomy	14 (1)	0 (0)	0.57
- Lactate – max. (mmol/l)	1.7 (1.2–2.6)	4.8 (2.5–9.1)	< 0.001
- pH - nadir	7.36 (7.30–7.43)	7.18 (7.05–7.31)	< 0.001
Outcome			
- Duration ICU stay (days)	1.6 (0.9–3.4)	2.3 (0.6–6.0)	0.92
- Duration hospital stay (days)	11.0 (7.0–16.7)	7.0 (1.9–14.0)	< 0.05
- Died in ICU	179 (17)	22 (88)	< 0.001
- Died in hospital	306 (28)	25 (100)	< 0.001

Data are expressed as n (%) or median (interquartile range – IQR 25/75%)

*Abbreviations*: kg, kilogram; cm, centimeter, SAPS, simplified acute physiology score; SOFA, sequential organ failure assessment; pts, points; MV, mechanical ventilation; ICU, intensive care unit; adm, admission; CA, cardiac arrest; HR, heart rate; MAP, mean arterial pressure;

**Table 3:** ICU-CA characteristics

Variables	ICU-CA *(n = 25)*
Time - ICU admission to ICU-CA (days)	0.5 (0.2–3.2)
Cause of Cardiac Arrest	
- Presumed cardiac	16 (64)
- Non cardiac	9 (36)
Cardiac arrest witnessed	25 (100)
Resuscitation Times	
- No-Flow (min)	0 (0–0)
- Total Resuscitation Time (min)	10 (2–15)
ROSC	17 (68)
Cardiac Re-Arrest	6 (24)
Mechanical chest compression device	2 (8)
Initial Rhythm	
- Shockable Rhythm	5 (20)
- Non-Shockable Rhythm	20 (80)
Defibrillation	5 (20)
Epinephrine	21 (84)
Epinephrine Cumulative Dose (mg)	3 (1–4)
Cardiac Failure post CA	10 (40)
Post-CA Shock	10 (40)
Temperature control	3 (12)
CPC – ICU discharge	
- CPC 1–2	1 (4)
- CPC 3–5	24 (96)

Data are expressed as n (%) or median (interquartile range – IQR 25/75%)

*Abbreviations*: CA, cardiac arrest; min, minutes; CPC, cerebral performance categories; mg, milligram;

### Cardiac arrest characteristics

The median duration from ICU admission to ICU-CA was 0.5 days (0.2–3.2). The initial cardiac rhythm was shockable (ventricular tachycardia/fibrillation) in 20% (*n* = 5) and treated by defibrillation. The median total resuscitation time was 10 (2–15) minutes. Sustained ROSC was observed in 68% (*n* = 17), and cardiac re-arrest was observed in 24% (*n* = 6). A mechanical chest compression device was used in 8% (*n* = 2). Aetiology of the ICU-CA was presumed cardiac in 64% (*n* = 16). 84% (*n* = 21) received epinephrine during cardiac arrest. Temperature control was initiated in 12% (*n* = 3).

### Outcomes after ICU and hospital stay

**Fig. 2:**
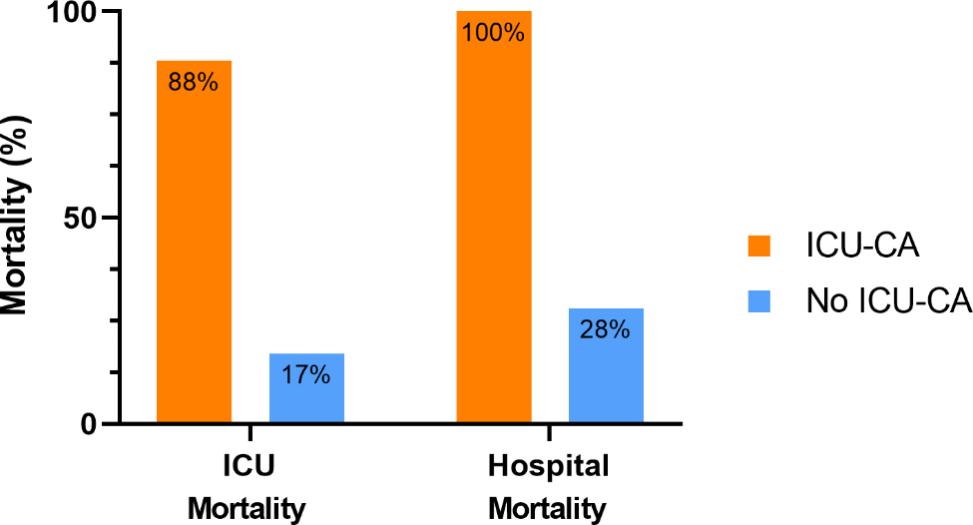
ICU- and Hospital mortality stratified according presence of ICU-CA

The overall ICU and hospital mortality rates were of 18% (*n* = 201) and 30% (*n* = 331), respectively. Accordingly, in patients with ICU-CA, ICU and hospital mortality rates were 88% (*n* = 22) and 100% (*n* = 25), respectively, hence, being significantly worse than in those without ICU-CA (ICU mortality: 17% [*n* = 179], hospital mortality: 28% [*n* = 306], both *p* < 0.001, see Fig. 2). The CPC at ICU discharge after ICU-CA was favourable in only one of the 25 patients.

## Discussion

In this large cohort of very elderly critically ill patients treated at a tertiary care ICU in Germany we found that 2% suffered a CA during their ICU stay. Severity of illness, vasopressor therapy and requirement IMV were associated with occurrence of ICU-CA. Of interest, patients with ICU-CA exhibited a 100% hospital mortality. To our knowledge, this is the first investigation of ICU-CA in the age group of critically ill patients ≥ 90 years.

Cardiac arrest occurring in the ICU represents a specific subgroup IHCA, as consequence of the distinct medical response and should considered as seperate entity of IHCA [15]. The reported incidence of ICU-CA in the literature exhibits considerable variation, ranging from 4 to 78 cases per 1,000 ICU admissions [11, 14–17, 26–28]. However, more recent studies have indicated a decline in these rates. Specifically, two recent prospective studies in mixed ICU cohorts observed an incidence of 22 to 23/1,000 ICU admissions [11, 17], a trend that may reflect advancements in the management and treatment of critically ill patients [15]. Notably, the highest incidences ICU-CA were observed in patients with underlying malignant oncologic conditions [29]. This variation in incidence rates can also be attributed to the highly heterogenic nature of these study cohorts. During the COVID-19 pandemic, some studies reported a noted shift from non-ICU-IHCA to ICU-CA [13]. As our study concluded before the onset of the pandemic, it does not provide insides into this specific period. While there seems to be a plausible association with the pandemic (e.g., less hospital admission, increased mortality before presentation a hospital or healthcare services), it is important to note that a definitive trend cannot be established and warrants further investigation.

In the current cohort we observed ICU-CA in 2% of the critically ill very elderly admitted to the hospital. This translates to an incidence 23 ICU-CA per 1,000 ICU admissions. This rate aligns with the findings from recent research on ICU-CA [11, 17, 27]. Earlier studies showed that about one-third of patients with ICU-CA are ≥ 75 years [17]. This finding is particularly noteworthy, considering the strong correlation of CA and age [30].

Comparing clinical characteristics, we found that about 60% of patients with ICU-CA were female. This can be attributed to the fact that there are significantly more female individuals in the very elderly age group due to demographic development. Most of the ICU-CA occurred in the first days after ICU admission, aligning with findings of previous studies on the topic [11, 17]. The majority of patients had an initial non-shockable rhythm and the presumed cause was mainly cardiac which is also in line with earlier studies [11, 17]. Furthermore, we found that only 12% were treated with hypothermic temperature control after ICU-CA. Although the effect of hypothermic temperature control in patients with IHCA was neutral patients should at least receive fever prevention [31–34]. If the low rate of hypothermic temperature control is attributable to the relatively low resuscitation time or can be explained by other factors remains unknown. However, the low rate found in the current study is in accordance with earlier prospective observational studies on the topic and should be investigated further [11, 17].

Organ dysfunction and organ failure after CA is frequently observed [35–40]. The high morbidity and mortality after CA were shown to be mainly triggered by post-CA shock and brain injury [38, 41]. The effects of pre-existing organ dysfunction and organ support are less clear. In our cohort, more than 90% of patients had invasive or non-invasive respiratory support at the time of ICU-CA. One large study found that mechanical ventilation at the time of CA is associated with noticeably decreased survival [42]. However, we did not observe similar differences regarding survival in this cohort. About 80% of patients had vasopressor support in place at the time of ICU-CA; we observed an association with an unfavourable outcome. This is in line with two previous studies which also found an association with the pre-arrest use of vasopressors with unfavourable outcome [43, 44]. About one-fourth of the patients received RRT prior to ICU-CA and RRT was first initiated in 42% of patients after ICU-CA. We did not observe an association with unfavourable outcome as described in earlier studies on cohorts of OHCA patients [36]. In our study we observed that 68% of patients with ICU-CA achieved ROSC, however, still leading to an ICU mortality rate of 88%. None survived until hospital discharge.

Of interest, loss of autonomy and unfavourable neurological status is one of the most feared status by elderly patients [45], which is contrast to the rate of observed favourable neurologic outcome in this study. Therefore, therapy should be restricted to those measures that are likely to lead to an acceptable quality of life with a unimpaired cognitive function [46]. This goal may be hard to achieve in patients with ICU-CA, and those who already suffer severe acute illness, as reflected by high SAPS II and SOFA scores and may additionally require IMV and vasopressor therapy. Limiting therapy in patients presents a significant challenge, particularly in those whose admission to the ICU seemed justified, despite advanced age. This is further complicated in those that were in relatively good health until the onset of acute illness; this was also observed in our study with a surprisingly low CCI. Careful consideration of medical indication and moral values of the patient and their relatives should be taken; however, this may be challenging in these situations due to various reasons. International guidelines provide recommendations for ethical and end of life decisions and underline the patient’s autonomy as one of the key points [47]. Therefore, patient’s wishes regarding medical care and therapy should be assessed and discussed with the patient and the family as early as possible if the clinical situation deteriorates, regardless of a potentially justified admission to the ICU. Although useful, in only 15% (ICU-CA: 8%, No-ICU-CA: 15%) of patients was an advance directive on site or deposited at the hospital which relevantly influences treatment intensity. The advance directive rates align with earlier observational data [48]. Furthermore, the pre-morbid status has to be taken into account in the elderly. As we observed high ICU mortality rates, providing assurances that care will remain appropriate, and address the wishes and moral values of the patient and their relatives can optimise compassionate care while avoiding futile life-sustaining interventions. Due to this measures many of ICU-CA can be anticipated in advance to provide appropriate, ideally personalized, care in such a situation.

This study has several limitations: First, the sample size of patients with ICU-CA in this study is low and, therefore, the conclusions are limited. However, this is the first and most comprehensive study on ICU-CA in this specific patient population. Second, this study included only patients with attempted CPR following CA, which should be taken into account when interpreting the results. We did not study patients in which CPR was not attempted as a consequence of predefined decision to limit intensive care based on medical judgement and patient wishes. Third, we presented retrospectively collected results on patients in a single (experienced cardiac arrest) centre. Thus, our results may not generally be transferable to other settings. Fourth, this study encloses a time period of eleven years. We cannot exclude that unmeasured time-dependent changes in treatment protocols may have influenced our findings. Sixth, residual confounding from unmeasured covariables is a matter of concern and cannot be entirely excluded.

## Conclusion

The occurrence of ICU-CA in critically ill patients with advanced age (≥ 90 years) is relatively rare. The observed mortality in the ICU and hospital was exceedingly high. Notably, providing cardiopulmonary resuscitation (CPR) did not result in any patient being discharged alive from the hospital. For very elderly patients, where ICU admission is deemed justified following thorough evaluation by experienced medical staff, supportive care should be prioritised. However, our findings suggest that administering CPR in these cases may not be advisable. Nevertheless, decisions regarding for or against advanced life support must be personalized.

## Electronic supplementary material

Below is the link to the electronic supplementary material.

Supplementary Material 1

## Data Availability

No datasets were generated or analysed during the current study.

## Abbreviations

kg: Kilogram
m: Meter, SAPS, simplified acute physiology score
SOFA: Sequential organ failure assessment
pts: Points
MV: Mechanical ventilation
ICU: Intensive care unit
