# Supplementary material for: Intensive care unit cardiac arrest among very elderly critically ill patients – is cardiopulmonary resuscitation justified?
Source: Scand J Trauma Resusc Emerg Med. 2024 Sep 11;32:84. doi: 10.1186/s13049-024-01259-1 (PMC11389322; doi:10.1186/s13049-024-01259-1)
Supplement: Supplementary file 1 — Supplementary Material 1 [file 13049_2024_1259_MOESM1_ESM.docx]

Supp Table 1 – Pre-existing comorbidities patients with and without intensive care unit cardiac arrest

| *Parameters* | *Non ICU-CA*  *(n = 1083)* | *ICU-CA*  *(n = 25)* | *p*-value |
| --- | --- | --- | --- |
| Cerebral arterial disease *n (%)* | 170 (16) | 1 (4) | 0.11 |
| Chronic lung disease *n (%)* | 86 (8) | 3 (12) | 0.46 |
| Chronic kidney disease *n (%)* | 104 (10) | 7 (28) | < 0.01 |
| Congestive heart disease *n (%)* | 234 (22) | 8 (32) | 0.21 |
| Connective tissue disease *n (%)* | 3 (0) | 0 (0) | 0.79 |
| Coronary heart disease *n (%)* | 165 (15) | 5 (20) | 0.51 |
| Dementia *n (%)* | 208 (19) | 0 (0) | < 0.05 |
| Diabetes Mellitus *n (%)* | 147 (14) | 3 (12) | 0.82 |
| Liver cirrhosis *n (%)* | 7 (1) | 1 (4) | 0.05 |
| Peripheral arterial disease *n (%)* | 100 (9) | 1 (4) | 0.37 |
| Malignancy *n (%)*  Solid tumor  Leukemia  Lymphoma  Solid tumor with metastases | 104 (10)  6 (1)  9 (1)  36 (3) | 1 (4)  0 (0)  0 (0)  1 (4) | 0.34  0.71  0.65  0.85 |

*Data are expressed as n (%)
Abbreviations:* AIDS, acquired immune deficiency syndrome; n, number; ICU, intensive care unit; CA, cardiac arrest;
